# Applying the 3Rs: A Case Study on Evidence and Perceptions Relating to Rat Cage Height in the UK

**DOI:** 10.3390/ani9121104

**Published:** 2019-12-09

**Authors:** Hibba Mazhary, Penny Hawkins

**Affiliations:** 1 School of Geography and the Environment, University of Oxford, Oxford OX1 3QY, UK; 2 Research Animals Department, RSPCA, Wilberforce Way, Southwater, West Sussex RH13 9RS, UK; penny.hawkins@rspca.org.uk

**Keywords:** animal welfare, rat, animal husbandry, refinement, 3Rs, laboratory animals, rat welfare, anthropomorphism, behaviour change, evidence

## Abstract

**Simple Summary:**

The use of animals in research and testing in the UK is regulated by the Animals (Scientific Procedures) Act 1986, which sets out how animal experiments must be licensed and regulated. Within this, the Code of Practice currently allows laboratory rats to be housed in cages that are 20 cm high, even though adults can rear up to 30 cm. Most adult rats therefore cannot stand upright in ‘standard’ cages. There is evidence that the ability to stand and rear up on the hind legs is important for rat welfare and enables them to explore their environment. Rats unable to stand on their hind legs in ‘standard’ cages will compensate for this with increased lateral stretching, which could indicate discomfort due to the inability to stretch fully and to exercise. Some animal research establishments in the industry recognise that housing rats so that they cannot stand up is a welfare (and scientific) issue, and have already invested in higher cages that allow rats to rear to their full height. Others, however, have not, citing a number of health and safety, financial, animal welfare, and scientific concerns. By surveying a number of different establishments, we identified the main barriers to adopting higher cages and ways to overcome them.

**Abstract:**

This article investigates the barriers to implementing higher caging in animal research establishments in the UK. The use of animals in research and testing in the UK is regulated by the Animals (Scientific Procedures) Act 1986, which sets out how animal experiments must be licensed and regulated. Within this, the Code of Practice currently allows laboratory rats to be housed in cages that are 20 cm high, even though adults can rear up to 30 cm. Most adult rats therefore cannot stand upright in ‘standard’ cages. We found that the main factors hindering the implementation of higher caging were classified into five different groups; health and safety, financial, animal welfare, scientific, and ‘human’. Suggestions to overcome these barriers are provided, as well as alternative animal welfare changes that can be put into place. We conclude that much of the desired evidence for moving to higher cages is already available, and therefore the focus should be on education and improving access to the existing evidence, in order to encourage facilities to work around existing financial and health and safety concerns.

## 1. Introduction

The 3Rs principle for humane experimental technique includes the concept of Refinement, i.e., implementing measures that will reduce suffering and improve welfare throughout an animal’s life. Refining housing, husbandry and care is an effective way of reducing stress, facilitating appropriate natural behaviours, and striving to allow animals to have a ‘good life’ in the laboratory [1,2]. Standards for housing, husbandry and care are generally based on a combination of current ‘good practice’ and evidence from animal behaviour and welfare science, where the latter exists [3,4,5].

Many guidelines and Codes of Practice make it clear that they (i) define *minimum* standards, which can and should be improved upon, and (ii) can be updated if new evidence about animals’ welfare needs becomes available [5,6]. With reference to (i), establishments are free to research the literature and use it as a basis for improving their own standards, evaluating refinements in-house if necessary.

However, although empirical research should—by definition—be objective, the interpretation of the literature and its implications may not be. Housing standards are usually defined by working groups comprising multiple stakeholders with differing priorities, so the final recommendations are likely to represent a compromise between the needs of animals and humans. At an individual level, personal beliefs and values will combine with economic, practical and scientific factors when individuals decide whether or not evidence is compelling. Understanding the types, and amount, of evidence that people require before they will change practice is thus critical to furthering the implementation of the 3Rs and in guiding research directions and communication.

**Figure 1:**
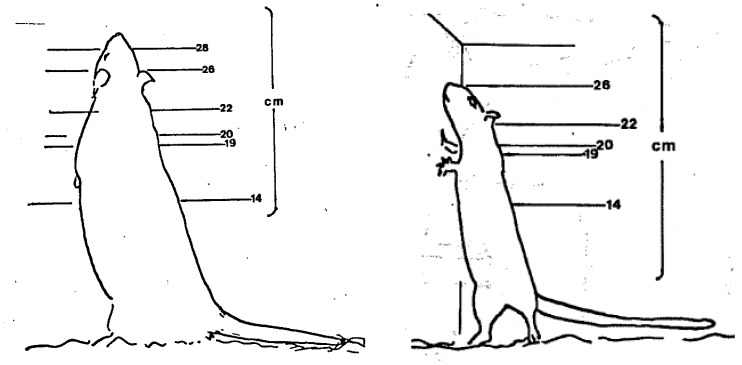
Left: 16 month old male Sprague Dawley rat in a bipedal posture, against examples of different cage heights. Right: rat rearing to over 26 cm at 8 weeks old (illustrations: Universities Federation for Animal Welfare).

This paper aims to provide some insights into perceptions of ‘evidence’ by focusing on how evidence has been, and is, used and interpreted to determine a single standard: the minimum cage height for laboratory rats in the United Kingdom. As this is a case study, it was pertinent to focus on a single standard in one jurisdiction, but the approach can be applied globally and used to reflect on how all ‘engineering’ standards are decided. Many Codes of Practice and standards for housing, husbandry and care (e.g., European Union, UK and US) do not permit adult rats to adopt an upright rearing posture [3,5,6]. For example, the European Union (EU) Directive 2010/63 requires a minimum cage height of 18 cm [6], whereas a standing adult rat can be 30 cm tall (Figure 1).

The EU standard was set out on the basis of advice from the Council of Europe’s Group of Experts on Rodents and Rabbits, which was tasked with defining housing that would meet the behavioural and physical needs of the animals [4]. Text from the Group’s background document is reproduced in Box 1.

Box 1 **Discussion of the literature relating to rat cage height by the Council of Europe’s Group of Experts on Rodents and Rabbits [4].**

- Rats regularly stand up-right for both exploration (Büttner, 1993) and social behaviour (e.g., boxing position, Nagel and Stauffacher, 1994).
- To determine minimum cage sizes for rats, the minimum spatial enrichment objects as well as the rats’ need for rearing up should be taken into account (Büttner, 1993; Ernst, 1994; Lawlor, 1990; Weiss and Taylor, 1985).
- A series of studies have shown that rats prefer cage heights of 18–20 cm (Büttner, 1993; Lawlor, 1983; 1990; Weiss and Taylor, 1985), but it has also been shown that rats spend most of their time in burrows if given the choice (Boice, 1977). Thus, the lid on a rat cage should allow enough height for both grooming, i.e., performing face washing while sitting upright on their hind legs, as well as withdrawal into lowered parts, for example underneath the food trough (Blom et al., 1995).
- The group considers that there is a scientifically valid basis for raising the minimum demands for cage heights for rats to 18 cm *.

* From 14 cm.

Some of these references are not digitised and no longer available, but we have been able to access Boice, 1977 [7]; Buttner, 1993 [8]; and Blom et al., 1995 [9]. Boice (1977) describes a series of experiments in which ‘albino’ rats were housed in an outdoor pen (in a precursor to the later ‘Ratlife’ experiment [10]). They rapidly began digging burrows with chambers, but the paper does not in fact present any time budgets or include any preference, or choice, tests. Büttner (1993) concludes that cage heights that allow rats to stand are used ‘intensively’ by adult animals, corresponding to 8.7% or 5.5% (depending on strain) of the total locomotor activity. Contrary to the citation in the group’s report, Büttner does not include any preference tests [8].

A preference test paradigm was used by Blom et al. to compare cages that were 8, 16, 24 and 32 cm high, all leading from a central cage. It reports that both sexes of rat spent time in all of the cages. Dwelling times in the 32 cm cages were 21.6% ± 3.8% for females, and 33.5% ± 7.4% for males, but there were no significant differences between the times spent in the four different height cages or between the sexes. The paper concluded that a cage of variable height, that included space for rearing and a low, darkened shelter, would probably be most attractive to rats [9].

The interpretation of these papers is of interest. The statement that rats ‘prefer’ cage heights of 18 to 20 cm does not seem to be supported by the references that we could access [8,9]. Blom et al. is used in the Council of Europe document to suggest that rats need to withdraw into lower areas, whereas it actually concludes that both lower and higher areas would be needed. Furthermore, setting aside the interpretation of all the papers, **four or five publications were taken to be sufficient evidence to justify increasing rat cage height [4]**.

The UK Code of Practice, published in 2014, includes a minimum cage height of 18 cm for rats, increasing to 20 cm for animals weighing over 250 g [5]. This is the same as the minimum cage heights for rats in the previous UK Code of Practice [11], which were taken from guidelines produced by the Royal Society and the Universities Federation for Animal Welfare (UFAW) [12]. The RS/UFAW guidelines were based on European standards, with the proviso that ‘where they differ it is to take account of good current British practice’. Article 2 of the EU Directive permits Member States to retain higher standards that were already in place, such as rat cage height [6].

Since the EU and UK standards were published, the literature on evaluating the impact of rat cage height has increased (Table 1). There is evidence that housing in cages that permit upright standing leads to reduced anxiety and stress, positive welfare and increased natural behaviours including exploratory behaviour [13,14,15,16]. It is important to recognise that wild-type behaviour is often conserved in domestic animals if they have the opportunity to express this [7,17,18].

Some establishments do now house rats in enclosures that allow them to stand upright, and higher cages are commercially available, but this is not yet standard practice. Discussions with the scientific community have indicated a number of reasons for this, relating to concerns about human health and safety, available resources, animal welfare concerns, scientific concerns, and perceptions that the animal welfare benefits of higher cages are not proven (PH, pers. obs.).

The existence of the new evidence set out in Table 1, and the variation in practice, suggested that a social science approach would be helpful in exploring these concerns more fully and understanding perceptions of what constitutes robust evidence that would justify changing practice.

## 2. Materials and Methods

One of the researchers (H.M.) visited eight different establishments representing seven different organisations (two establishments, CRO A 1 and CRO A 2, were associated with one organisation). We endeavoured to obtain a representative sample of the different types of organisations that house laboratory rats: academic (universities), commercial (pharmaceutical companies, Contract Research Organisations (CROs)), and governmental. The organisations consisted of three universities, two pharmaceutical companies, one CRO and one governmental department (see Appendix A for a full list of facilities and participants). Organisations were recruited by emailing a project brief to existing contacts in animal research facilities across the UK. A call for participants was also circulated in relevant laboratory animal newsletters, to which some of the organisations responded.

Semi-structured interviews (see Appendix B for a detailed interview guide) were conducted. Questions were focused on participants’ experiences of the barriers and solutions to increasing cage height in their own facilities, as well as on their perceptions of rat cage height in the wider industry. The researcher was also given a tour of the rat housing at each of the facilities. At each establishment, a number of people were interviewed, including Establishment Licence Holders (ELH), Facility Managers, animal technicians (AT) (also known as animal technologists), Named Animal Care and Welfare Officers (NACWO; senior animal technologists), Animal Welfare and Ethical Review Body (AWERB; local ethics committee) chairs, Named Veterinary Surgeons (NVS; the attending veterinarian) and scientists. A total of 37 individuals were interviewed, some individually and some in groups of two or three, with each interview lasting approximately 45 min to one hour. Interviews were audio-recorded then transcribed to ensure accuracy. Transcripts were then analysed to identify the main categories of obstacles that were cited to increasing rat cage height across multiple interviews, and quotations were subsequently sorted into these categories such as scientific and financial.

Ethical considerations were observed, including informed consent from all participants and anonymisation of individuals and organisations. Participating organisations are labelled in this report by the type of institution followed by a letter.

## 3. Results

Out of the eight facilities visited, three (University A, Pharmaceutical A and CRO A 2) used exclusively 20–23 cm high rat caging, one used only 30 cm caging (CRO A 1), and the remaining four used a mixture of higher and lower cages. Of the mixed facilities, two used cages higher than 30 cm, the commercially available double-height model, 65 cm (Facility A) and 46 cm (Pharmaceutical A) high marmoset-style cages respectively. This section begins by outlining the main challenges and obstacles to introducing higher caging—health and safety, financial, animal welfare, scientific, and human perceptions—as well as strategies to overcome them. There is subsequently some discussion of the process of enacting change and influencing attitudes.

### 3.1. Health and Safety Issues

Interviewees frequently expressed that higher cages were bulkier and therefore more difficult to handle:

“If you’re housing in fours you’ve already got a couple of kilos worth of animals plus the weight of your cage. And nobody’s truly come up with the ergonomic way of doing it.”

(Facility Manager, University A)

Specifically, there was a concern that as higher cages would require taller racks, lifting cages from the top and bottom shelves could present manual handling issues and risk of repetitive strain injury. There were several common strategies employed by facilities to overcome these health and safety challenges. At Pharmaceutical A, where they used mostly standard cages but also tower rack cages (46 cm height), they used a robotic cage wash to assist with cleaning the cages, although that in itself presented complications for larger cages because the tower rack cages had to be dismantled before entering the cage wash.

Appropriate manual handling training specific to larger cages was also cited as a strategy. Some facilities also avoided using the top and bottom shelves of the racks (so as to avoid lifting the cages from either very high or very low positions), although this very much depended on having sufficient space which was not always available. The facilities also limited the group numbers to two to four rats per cage (less than the manufacturer’s recommended maximum) to minimise weight issues. A common strategy was also to remove water bottles before lifting the cages.

Depending on the cage model, bigger cages may not present heightened health and safety concerns. At Pharmaceutical A, which had marmoset-style cages on wheels, the health and safety assessment concluded that if the cages were being handled appropriately there was actually a lower risk of injury than handling standard caging because fewer bending, twisting or lifting motions were required.

One way to anticipate and address health and safety challenges was encouraging technicians to voice their concerns. For instance, the Health and Safety Officer at Pharmaceutical A referred to “a ‘speak-up’ value” in their organisational culture. This is a crucial approach as animal technologists are key players in enacting change, as will be further explored in Section 3.6.3.

### 3.2. Financial Issues

Five out of eight facilities cited financial concerns as a significant barrier to purchasing higher caging. Several strategies were employed by different establishments to alleviate these and tended to differ depending on the type of institution. For universities, whose projects are mostly funded by grants from funding bodies, one researcher suggested working in the costing of higher cages at the grant application stage. This would include checking projected rat housing and general rat running costs and writing them into the grant. A researcher from University C identified a tendency in the scientific researcher community to fail to factor in rat housing and animal welfare at this stage, stating that “Researchers that use animals aren’t always conscientious enough at making sure all their animal welfare and housing needs are covered in the budget”. This researcher suggested that the animal house could be clearer and stricter regarding rat housing standards, thereby incentivising researchers to budget for higher running costs to ensure better welfare.

Facility A made acquiring new, higher cages more financially feasible by gradually purchasing 30 cages over the course of two years. Furthermore, they deliberately designed the cages to be suitable for multispecies use, such as rabbits and ferrets, so were able to demonstrate added value when making a case to capital expenditure.

Another avenue for minimising the cost of moving to higher caging is seeking collaborations with laboratory animal equipment manufacturers. University B approached a prominent manufacturer to request financial assistance with a trial for higher caging, who agreed to contribute towards operating but not equipment costs, which was reportedly beneficial to the suppliers’ reputation and their working relationship with the university. This also allowed for faster and more effective testing of the suppliers’ products and significant saving on trial costs:

“When you have funding for studies the most expensive thing always is the personnel time. It’s not a cost here because we have interested, willing enthusiastic staff willing to contribute time on top of their normal duties. For a bedding trial with four or five people and several sites you’re talking a million pounds type study to run that over 12–18 months. So actually the cost is very low in comparison here.”

(AWERB Chair, University B)

Space constraints featured quite often in discussions around financial concerns, and in some facilities they were particularly acute. Mostly space was seen as an immovable barrier, and aside from building a new facility, other solutions were not mentioned. One scientist, however, saw space constraints not as an unchangeable obstacle, but a reflection of the poor scientific quality of studies, arguing that the community should be focusing more on refining scientific models, as that would reduce the number of animals needed for studies, and thereby make space less of an issue:

“The number of animals that we use here has plummeted. One of the reasons the number of animals has reduced so much is because we have refined our models, not just because we are doing less work...The way around (space constraints) is to refine models and design better studies.”

(Scientist, Pharmaceutical A)

Ultimately however, in all organisations, the rhetoric advocated was that if the animal welfare benefit was sufficiently significant and evident, finances would not be a barrier:

“Obviously financing is important for big companies, but when you talk to them, most of them will say financing is not an issue when it comes to animal welfare.”

(Scientist, Pharmaceutical A)

There was also a consistent stated drive to improve upon animal welfare, with an animal technologist from University B stating that “Even with our small room, cost constraints and budget constraints, there is *always* something we can do to improve animal welfare.”

### 3.3. Animal Welfare Issues

The majority of interviewees did not cite animal welfare concerns regarding introducing higher cages, although some mentioned a concern that animals housed in bigger cages after surgery could be more likely to damage sutures by climbing. Similarly, Facility A housed rats with vascular access buttons on their backs and had been concerned that the rats would knock the ports in larger cages with more opportunities to climb and be active. They controlled this risk by monitoring the rats and providing softer enrichment such as fabric hammocks.

Some argued that rats preferred small spaces and therefore a larger cage would create more stress, with a scientist from University A stating that “rats tend to migrate towards the smallest darkest space…so I’m not sure of the benefits of a larger space”. However, a scientist from University C, a higher cage facility, argued that rats preferred small dark spaces to open spaces in behavioural tests, but “only with a novel environment that isn’t familiar”. This statement was tempered with the caveat that the level of stress in larger spaces depended on the age of the rat and their exposure to bigger cages:

“…you wouldn’t want to take a year-old rat and suddenly put it in a new environment because when they’re older they’re a lot less behaviourally flexible in terms of adapting to new spaces, not just physically but cognitively as well so they’ll get more anxiety because they won’t recognise things. So I would say you would want to introduce it from weaning.”

(Scientist, University C)

Similarly, there was an animal welfare concern cited for facilities with short studies, due to rats’ perceived neophobia:

“If they are only in the facility for five days, if they come from breeders where they have been kept in small cages all their life, and you suddenly put them in a large cage, it actually could be more stressful for them…If they are in the facility for more than a week, that gives them more than enough time to acclimatise to new conditions and enjoy the benefits.”

(Scientist, Pharmaceutical A)

There was also a concern voiced about adverse effects on aggression.

“I don’t know whether having different areas in the cage leads to more territorial behaviour. If you put them in a small enough cage, there are some animals that you can just force shared cooperativity on them. If you give them more space they’ll say, ‘this is my corner and actually now I’m going to fight to defend that corner because I know there is enough space for you in that corner.’”

(Scientist, Pharmaceutical B)

Other factors that constrained moves to higher caging related to animal welfare concerns about commercially available, double-height 30 cm high individually ventilated caging. Participants in six out of eight facilities raised issues with the reduction of floor space on the bottom and the fact that the rats did not have a large space in which to rear:

“I think the (double-height cages) are kind of a loop-hole. There is not a lot of rear space because of the platform around the back. Not all rats in the cage can stand up at the same time.”

(AT, University B)

However, there was acknowledgement that double-height IVCs (Individually Ventilated Cages) could be suitable for certain uses in space-constrained areas. Staff at Facility A, where 65 cm high marmoset-style cages for rats were used, acknowledged that in other parts of the business these cages would be an appropriate solution:

“…we are looking into providing more height within infectious disease models that need biocontainment. One consideration is to use one of the commercially available double-decker style cages in that area.”

(AT, Facility A)

### 3.4. Scientific Issues

Most respondents did not have scientific concerns as long as all the variables were kept consistent within a study. For instance, a scientist from University C declared “I’m always really supportive of enrichment changes to my animals but it has to be all of them.”

The scientific issues tended to vary depending on the type of study being carried out. A practical scientific concern related to studies involving biotelemetry transmitters or cameras. Some respondents stated that in IVC double height cages, the telemetry signal could not reach through the cage wall and both floors (this was not an issue for another establishment using open top cages). People working on the Rodent Big Brother project [19] also reported that the camera could not reach the full height of a double height cage.

One repeated scientific concern was the worry that the baseline for studies would be shifted and therefore would undermine future studies. Establishing a new baseline of data could simply be the case of conducting one experiment to ensure that the new data with the new cages looked identical to the old data. The complication arose when new data were different, necessitating re-validation. Scientists from different facilities mostly agreed that a direct comparison study between smaller and larger cages would have to be conducted in order to validate the new cages. Some researchers saw the need to re-establish the baseline as something solely negative, consuming time and resources and distracting from the study. One researcher, however, found differences in baselines productive:

“I would think it was interesting and I would try to get some funding to do a proper study…If it was quite a common change that many places are dealing with like something about suspended tunnels, then I would look at literature... I think a lot of people would see it as a nuisance, but I would see it as interesting.”

(Scientist, University C)

Similarly, one researcher expressed the view that re-validation was a valuable part of progress, which would refine the model and potentially mean that fewer animals were used in the future:

“…you are re-validating to improve your model. Yes, you are using more animals in the meantime, but getting better science, using fewer animals and ultimately animals are kept in better conditions. It is a short-term cost for a long-term gain. And without that, you are never going to go forward at all.”

(Scientist, Pharmaceutical A)

Another researcher cited change as an inevitability of laboratory animal research and suggested that moving to higher cages was not so significant because unexpected changes often happened that required re-validation:

“…the nature of the business we’re in, things change. Things can change because the animal supplier doesn’t tell you they’ve changed the bedding or the feed...sometimes you just have to re-validate. We’re going to move buildings, we will have to check stock and make sure the models still behave in the same way...You have to check the stress of moving the animals, using a new environment with a new microbiome potentially, there’s all sorts of factors that can contribute.”

(Scientist, Pharmaceutical B)

A common claim was made that the scientific concerns of changing caging were much more significant for behavioural than for non-behavioural studies:

“If you were doing some very sensitive behavioural studies that relied on spatial processing, animals that have been kept in 2D environment that are then placed in a 3D environment, I’d imagine that there would be some differences in their neurobiology in terms of their spatial hippocampus.”

(Scientist, Pharmaceutical B)

“The measures are very subtle in the pain field, and the things we’re looking for are very small, and if suddenly we are changing how a rat lives or spends most of its time, I’m not sure about the impact of that.”

(Scientist, University A)

This was countered by a behavioural researcher, who believed that there was a false dichotomy set up between behavioural and non-behavioural studies:

“I think if something is happening behaviourally, it is then therefore happening at all the levels below that because something has to change in the physiology to cause a change in behaviour. So, for other people it’s not that it’s not happening to your animal, you’re just not seeing it and you’re not measuring it.”

(Scientist, University C)

The refrain of ‘better science, better welfare’ was repeated throughout all the facilities visited, with most asserting that a more naturally behaving model was scientifically superior. There was rhetorically little disconnect set up between science and animal welfare, and the two were seen to be very complementary, although this was not always maintained in practice.

“...our stuff is supposed to be clinically applicable and rats in cages I always think is a bit like looking at prisoners...it’s not actually a reflection of how the general population are so I feel like pushing our animals to be as naturalistic as possible is going to mean their brains, and the rest of their physiology is working in the most optimal and naturalistic way so what we’re studying is more likely to be translatable to humans.”

(Scientist, University C)

One researcher saw the disconnect between welfare and science in practice as a result of poor scientific quality:

“…people are questioning the welfare, but they should be questioning the science. You should be asking ‘Why can’t you adapt your science to make this welfare change work?’ You have to go through scientific and ethical review for all studies, as part of those reviews we are supposed to be asking is this still a good model…Certain models are routinely used 40 years after they were first developed, and the translatability is very poor.”

(Scientist, Pharmaceutical A)

### 3.5. Human Attitudes

Participants expressed assumptions and perceptions relating to rat behaviour, including the need to rear, and how humans can comprehend animals’ needs. Some of these could act as ‘human’ barriers to implementing refinements.

#### 3.5.1. Perceptions Relating to Rearing Behaviour

All facilities, even those with standard height cages, claimed that their rats could rear fully upright and did not observe rats being unable to do so. Furthermore, some individuals at University A expressed scepticism about the importance of rearing in rats’ behavioural repertoire. In a similar vein, one AWERB Chair, when discussing strain differences between rats, disputed that rearing was universal:

“A rat isn’t a rat. So if you walk into a rat facility, you’ll find that there are strain differences with how eager a rat is to actually rear up and peer. Rats are very intelligent creatures and they’re quite interested so if you had a hooded rat, you would almost always come up and peer. If you had a Sprague Dawley, some of them would but not all of them so it would be a mistake to assume that all rats always rear up.”

(AWERB Chair, University C)

#### 3.5.2. Wild Rats versus Laboratory Rats

Some respondents cited wild rat behaviours as proof that rearing was not a significant part of rats’ behavioural repertoire, such as this exchange:

“I do have some wild rats in the garden...they do spend a lot of time around the birdfeeder but it tends to be running and scurrying so I would think…how much do they actually spend being able to rear up completely?”

(Biomedical Research Facility Manager, University A)

“We have a big birdfeeder and you can see the (wild) rats clamber up the pole. They’re not standing there, rearing up, they’re using it as a climbing implement. And unless, in these cages there is some kind of climbing frame and some way the rats can use that and benefit from that height, I’m not sure that saying ‘so they can stand up on their rear legs’, I’m not sure that a rat would be necessarily doing that.”

(Scientist, University A)

However, some expressed doubt that wild rats were still a useful comparison with laboratory rats:

“We’ve also got to remember that the laboratory rat is quite a different beast from the wild rat.”

(Chairman, CRO A)

An animal technologist from Facility A argued that wild rats and laboratory rats were still a valid comparison, that wild rats did indeed express rearing and climbing behaviour, and these primary instincts were unchanged by the laboratory environment. They noted that laboratory rats would spontaneously climb, burrow and dig when transferred into larger cages that enabled these behaviours. In this facility, observing this increased behavioural repertoire in higher cages helped to improve attitudes towards the rats and staff morale:

“When you see animals exhibiting all these natural behaviours, you appreciate them more.”

(AT, Facility A)

“…the newer caging type has strengthened [the human-animal bond] because people interact with the rats more…Now when you go into the room you will find the rats respond to someone coming in by standing up to see what’s going on and wanting some Rice Krispies…We also hand-feed them more because with these cages it’s easier to do that. So you’re definitely more bonded because positive reinforcement with feeding is easier.”

(NVS, Facility A)

#### 3.5.3. Avoiding ‘Anthropomorphism’

Many interviewees were cautious not to conflate the needs of humans and those of animals. There was a conscious effort to identify and problematise anthropomorphism if it ever arose:

“The problem with some of the things we do in life, particularly in this arena, is we anthropomorphise animals, so we sort of look at it and think ‘I would like to stretch’.”

(Chairman, CRO A)

“I guess I’d want to know that if you were to make a change that the change was based on evidence rather than just making us as humans happier, that I can now as a researcher go into a facility and know my animal has run around this big cage.”

(Scientist, University A)

“You’re always saying, ‘This would be nice for the animals’. But do they really want it? There is a tendency to think, ‘If I were a rat I’d like that’.”

(AT, Pharmaceutical B)

Some saw this anthropomorphism as benign, and very important for improving the quality of the animal technicians’ working conditions, due to the ‘emotional labour’ they expend in caring for animals [20,21,22].

“…people think, ‘that’ll be nice for them, they can hide in that, or chew on that, or they’ll love to forage for these things.’ But then you’ll ask them what data are there to suggest that actually is producing a genuine improvement in their animal welfare, it’s harder to pin down…some of it does become a bit anthropomorphic, but actually for animal technicians who are working day to day with sick animals, animals that they kill, animals that they take blood samples from, feeling that they’re doing something positive for those animals is a real boon for them so if it does the animals no harm, but the animal technician feels that it’s making an improvement, then that’s making an improvement on the animal technician’s quality of their job experience and the animals they’re caring for.”

(Scientist, Pharmaceutical B)

### 3.6. Enacting Change

A general resistance to change was identified, as voiced by the Facility Manager at Pharmaceutical A, who observed that “change is not always accepted easily unless people are convinced it’s a good thing to do.” Therefore, in order to enact change the onus was placed upon the proponent of the change to produce persuasive evidence. With a view to thinking about how to change attitudes and facilitate refinement, we asked interviewees what kind of evidence would convince them to support a change to higher cages.

#### 3.6.1. Types of Evidence

There was a need across all facilities for ‘hard’, scientific evidence to justify change. Respondents mentioned several desired components of this evidence; firstly, such a study would need to monitor rats over a 24 h period due to many participants not having observed rats during their most active nocturnal period. Film footage of rats was also valued because many looked for evidence of how the rats used the space, as in the Rodent Big Brother project [19]. Moreover, the Biomedical Research Facility Manager at University A celebrated one study by Marcus Stauffacher exploring bone density and muscle growth in rabbits in smaller versus bigger cages [23], and stated that a study on rats using similar measures would be “conclusive”. This focus on having accurate measures was again entangled with the discussion of the fear of anthropomorphism. There were some suggested measures for studies such as anxiety levels and levels of stress hormones in rats in new versus old caging, but generally there was a lack of clarity about which measures to use in such studies to evaluate the benefits of higher caging:

“…there would be limited justification for trying to do any systematic study because what would your endpoint(s) be? Other than comparison of normal versus abnormal behaviour, it’s very difficult to assess whether an animal is happy or not...So how would you objectively compare two scenarios in which you had normal behaviour of the animals?”

(AWERB Chair, University C)

“So a lot of things is just preference, so the rats like this, but that’s not good enough because they might like it but you need to be able to measure the welfare improvements.”

(Scientist, University C)

The NVS from Facility A, when discussing what counted as compelling measures in a study, stated that “our focus is to stick to objective data on well-being where we can” and therefore visual observations of rats would not be sufficient, but instead they believed that a study that tested ultrasonic vocalisations of rats would be as these measures accurately indicated rats’ affective states. This was also seen as a way to divert concerns of anthropomorphism.

In order for the study to be translatable and convincing, scientists expressed a preference for it to be in a similar field to theirs. A behavioural scientist from Pharmaceutical A who specifically worked on stress models observed that studies varied greatly from model to model yet would consider non-stress models to be translatable as long as they were behavioural studies.

The practical and financial challenges inherent in setting up studies in terms of time and cost was also often enumerated:

“…studies would be very time-consuming and most places don’t usually have the luxury of saying come off routine duties and just sit there and do the study.”

(AWERB, University C)

#### 3.6.2. Anecdotal Evidence versus Scientific Studies

As well as empirical evidence, some level of the anecdotal and subjective evidence was compelling, as long as it was well-presented and argued:

“I personally think if you are going to make any improvement...you give me the data and you give me the evidence whether that’s subjective or whether that’s hard data…just show me and then prove to me the differences, prove to me what would be the benefit for myself as well as the animals.”

(Facility Manager, Pharmaceutical A)

However, this was the case for those already predisposed towards making an animal welfare change; a scientist from University C argued that “to convince people that are more sceptical…you need studies.” A researcher from Pharmaceutical A who had been using 46 cm high tower rack cages for four years decided to conduct a direct comparison study in order to convince sceptics, identifying himself as a former sceptic. In a similar vein, when an animal technician who was passionate about improving welfare for rats expressed a desire for larger cages, the NVS from Facility A encouraged him to obtain some objective data on rat well-being in different cage types as “our capital expenditure process expects requests to be evidence-based”.

#### 3.6.3. Drivers of Change

Facilities that had made the most progress in implementing animal welfare changes were driven forward by one or several charismatic individuals. In Pharmaceutical A, one scientist, with the support of other colleagues, had been chief in pushing for the purchase of tower rack caging. There was also much initiative taken by technicians to design cages and drive change:

“…for the rat caging project (AT) was really the instigator and driving force behind it. He came to me personally to express his concerns and ideas about rat caging because he was really passionate about it…his long-standing interest in whether rats could express behaviours in standard laboratory caging coming from keeping rats as pets and so being very familiar with the species.”

(NVS, Facility A)

Animal technicians were often very progressive and pushed for change, although at other times they were cited as the biggest opposition to change:

“Initially there was a lot of opposition from staff, but once they’d used them for two to three weeks, they began to like them. And once they’d seen the benefit for the animals, they were complete converts.”

(Scientist, Pharmaceutical A)

“We had them here to trial, the technicians were unsure about them at first, to gain their buy-in, it was important that we listened and empowered the staff to make changes to the cage design.”

(Facility Manager, Pharmaceutical A)

Animal technologists nevertheless were crucial to implementing change, as although ‘hard’ scientific evidence was desired, there was also much value and trust placed in their experience and opinions:

“I think the most compelling argument is when an animal technician says, ‘I don’t know what it is but there is something wrong with these animals, they’re just not quite right.’”

(AWERB Chair, University C)

“…it’s not always absolutely rock-solid, quantitative, statistical *p*-value…Some of it is actually, you just look at it and say this looks like a happy cage, faeces are all in one corner, the animals look relaxed and happy, are grooming, and you have to use that animal technician experience to assess it.”

(Scientist, Pharmaceutical B)

“We should be taking technicians’ ideas and developing them and should be giving them the time and resources to come up with ideas.”

(ELH, University C)

#### 3.6.4. Parallels with Mouse Tunnel Handling

Interviewees frequently compared the issue of rat cage height with that of mouse tunnel handling, seeing it as good practice for implementing a new welfare change. All facilities had at least trialled tunnel handling. This could be a test case to observe how a welfare change is successfully implemented and how attitudes are influenced.

“You look at the work on mouse tunnel handling…when Jane Hurst’s work first came out, people were very much not convinced by it but now she’s published more on it and there’s more evidence and now I would say there’s a real ground-swell of people.”

(AWERB Chair, Pharmaceutical A)

“The whole thing about cup-handling at the moment, they’ve done proper ethological studies to show picking mice up by their tails is aversive because of response to predators so a very simple demonstration that cupping or using tubing reduces stress and you can see changes in cortisol levels, so that’s the sort of data for me as a scientist, that’s what I want to get to.”

(Scientist, Pharmaceutical B)

There are also practices to be observed regarding persuading members of staff to support a welfare change. One member of staff from Pharmaceutical A cited mouse tunnel handling as a successful example of what she called a “two-pronged approach” to overcoming opposition:

“…I think it needs that two-pronged approach, I think it needs the gently gently, the here you are, here’s the evidence and then, sorry this is for the animals’ welfare it’s going to be better for our scientific results in the long-run, so you’re just going to have to get on with it…I think it takes for them [technicians] to be convinced from the inside but also told from the outside.”

(Facility Manager, Pharmaceutical A)

#### 3.6.5. Communication

Generally, the facilities housing a smaller number of rats found it easier to implement animal welfare changes; the former Facility Manager and NACWO at University C theorised that it was less difficult to carry out refinements because they were not a large rat user, but he imagined that places with thousands of rats would have significant financial and space constraints. Similarly, staff from Facility A suggested that the size of their organisation allowed a better culture of communication, facilitating animal welfare changes:

“Our philosophy is that if an evidence-based refinement is proposed, our default would be to adopt it, unless there is a specific reason not to. Again, it is far easier for us to work in this way as we are a small organisation.”

(NVS, Facility A)

“We’ve done it in a way that is right for us and how we’ve gone about it might not be right for another facility with thousands of rats.”

(AT, Facility A)

Interorganisational communication was also vital in expediting animal welfare changes, such as Pharmaceutical B soliciting advice from a university that had been using double-height cages for several years, and employees at Pharmaceutical A touring the tower rack caging at a different establishment. This transmission was facilitated by employees who moved from one organisation to another.

#### 3.6.6. In-House Evaluation Studies

Some establishments visited by HM had conducted their own in-house evaluations of higher rat cages. One researcher from Pharmaceutical A is working on a forthcoming publication that follows on from Makowska and Weary’s work [16], showing that in terms of behavioural science (excluding stress type models), there is no evidence of a negative impact on the quality of science produced by housing rats in larger enriched cages.

An in-house study at Pharmaceutical A compared rat behaviour and physiology in 22 cm high versus 46 cm high cages, with similar but greater enrichment in the latter housing condition. Rats in the higher cages showed increased exploratory behaviour, used a larger range of areas for nesting, had fewer antagonistic interactions and showed no evidence of anxiety from being housed in these higher cages. No difference was found in relative body fat ratios and measures of faecal corticosteroids. The study concluded that the rats housed in larger, enriched cages had better welfare. The establishment now uses higher cages in one of their facilities but not at the main facility, although the author of the in-house study is attempting to enact this change.

In an internal study at Facility A, weaned rats were housed in old marmoset caging to observe whether the larger caging affected their growth curve. The rats in the larger caging did reach the same size as the rats in the standard caging, but this took longer, presumably because they were more active. Some other internal studies have found behavioural differences between rats in higher and lower caging. A study from one of the facilities, presented to the UK NC3Rs (National Centre for the Replacement, Refinement and Reduction of Animals in Research) in 2016, measured the ultrasonic vocalisation of rats in marmoset cages versus standard cages. They observed that ultrasound data were consistent with increased well-being in the larger cages.

### 3.7. Beyond Rat Cage Height

It is important to observe that cage height is not the only animal welfare factor to consider in rat housing, and that many other factors were perceived to influence welfare, such as variety:

“It’s all about variety for the animal versus practicality for the people working with the animals...putting a little toy in is not going to cut it for very long. It’s variety, change of scene.”

(R&D Manager, In-Vivo Sciences, Pharmaceutical B)

In a similar vein, complexity was seen as central. Some individuals found the complexity of the cage to be more crucial than height:

“For rats, I think having complexity is more valuable…rats are very curious, whereas just having a height, having an additional platform for example, I don’t think addresses all of their needs.”

(Associate Director, In-Vivo Sciences, Pharmaceutical B)

#### Alternatives to Higher Caging

Although increasing rat cage height is crucial, some facilities were not able to make that change, or at least not immediately. However, there are still other animal welfare improvements that can be implemented. Two facilities made use of playpens which allowed rats to exercise for a fixed amount of time every week. CRO A, although they already used 30 cm double-height cages, used a playpen and Pharmaceutical B similarly held ‘Ratty Playtime’. University B were also considering playpens as a compromise to changing all their cages, which was not immediately feasible with their space constraints. Moreover, University C linked two of their 26 cm high cages together so rats could move in between them, allowing them more space and variety.

There is also a need to look beyond individual facilities and focus further back in the supply chain on animal breeders, especially since facilities who carried out short studies felt they were less able to make a meaningful welfare change:

“…in terms of that animal’s lifetime experience, the bigger question is what did they experience in the breeding colony of the animal supplier? If they’ve been conditioned to live in single-storey caging because that’s what the animal supplier does, it’s great that the final two weeks of their life, we’ve given them a great environment, but actually before that there was maybe six weeks…with the animal supplier.”

(Scientist, Pharmaceutical B)

## 4. Discussion

The survey provided helpful insights into participants’ understandings and assumptions about the animal welfare, resource and scientific implications of increasing rat cage height. This section examines the responses enumerated in the results section.

### 4.1. Resource Implications

On the basis of the interview responses, higher cages can present more manual handling issues than ‘standard’ cages if they are lifted and handled in the same way. Human health and safety are clearly important, yet the question remains of whether the fact that taller cages can be heavier is a justifiable reason not to increase cage height. Establishments using higher cages cited ways of reducing the risks to staff, such as by not using the top and bottom rows of a rack, housing fewer rats per cage, and removing water bottles before moving cages. These approaches potentially have resource implications, as more space, cages, or time will be required.

The financial resources for higher cages were obtained in several ways, for instance researchers can include these in costings within grant applications, and this could be driven by animal houses having clear expectations for refined rat housing. One establishment implemented a budget for replacing rat caging over a defined time period, also designing cages that could be adapted for other species (e.g., guinea pigs and rabbits) to maximize cost-effectiveness. A collaboration with a cage manufacturer, which funded some operating costs, was also cited as a way of overcoming financial constraints. Approaching manufacturers to solicit collaborations may however only be a strategy suited to larger establishments with the ability to leverage their reputation and variety of personnel. Respondents from establishments that were relatively small, in comparison with industry facilities, felt that it was easier for them to implement refinements such as higher cages. This was because communications were easier, and the absolute financial outlay was smaller. Alternative approaches such as rat playpens and linking cages are also ways of improving welfare with reduced financial outlay.

In addition, caging for rabbits, guinea pigs and ferrets is larger and heavier still. However, these cages are designed so that animals can be removed via doors in the front, without removing the cage from the racks. Dedicated trolleys are used to remove cages for cleaning. There are obviously economic costs associated with this, which would have to be considered against the benefits to animal welfare, staff morale and data quality.

#### Type of Caging

The type of caging is also likely to influence the decision to move to higher cages. What became clear from the establishment visits was that the debate about higher versus lower caging often became overshadowed by the comparisons between open-top cages versus IVCs. There was much doubt expressed about the efficacy of IVCs, which often was conflated with higher cages because the model that first came to mind to many respondents was the commercially available double height IVC. Having IVCs as opposed to open-top cages was seen by some interviewees as an extra barrier for cost and space because they required bulky specialised equipment such as air handling units. It also restricted the positioning of the cages, and the ability to make optimal use of a small space. Similarly, open tops allowed for more choice in ways to implement higher caging; University B had raised their cage height from 22 to 34 cm by adding a different higher lid, which would not have been possible with an IVC. Therefore, acknowledging that implementing higher cages does not necessarily require moving to IVCs would help to maintain the focus on rat cage height and welfare.

### 4.2. Animal Welfare

Points that were made regarding animal welfare could be divided broadly into practical issues—if study animals had special needs—and assumptions about rat behaviour and welfare needs.

An example of a practical concern was that animals housed in higher cages *might* damage sutures by climbing, but no evidence was provided for this. In contrast, the facility housing rats with dorsal vascular access buttons, which was concerned about rats in large cages knocking the ports controlled this risk by monitoring the rats and providing softer enrichment such as fabric hammocks. Establishments using commercially available double height cages also expressed some practical welfare concerns about these, stating that the floor area is small and there is insufficient space for the animals to all stand up at the same time.

Beliefs relating to rat behaviour and welfare included assumptions that larger cages would be stressful because rats are perceived to like small, dark spaces, or would lead to an increase in territorial behaviour. However, the literature demonstrates that larger cages benefit rat welfare, without problems arising from territorial behaviour [14,16,24,25,26]. A view was also expressed that rats would have to be housed in larger cages from ‘weaning’, because older animals would be less behaviourally flexible and more anxious. With respect to concerns about behavioural inflexibility, a study that involved housing rats with, and without, access to the top half of a 40.4 cm cage, alternating treatments over periods of several weeks, reported behaviour consistent with a positive affective state when rats were allowed access to the full cage height [14]. These assumptions may indicate a knowledge gap in some aspects of the animal behaviour and welfare literature, as recognised in a survey by a psychopharmacologist, which found that knowledge of animal behaviour was lacking in behavioural scientists [27].

Expressions of scepticism about the importance of rearing to rats were also of interest. Anecdotal evidence was presented of strain differences in the laboratory and observations of wild rats in people’s gardens, and respondents expressed assumptions that laboratory rat behaviour is very different from that of the wild rat. It is noteworthy that these isolated observations, perceptions and assumptions were cited as evidence that standing is not important to rats, in contrast to the robust evidence required by some respondents before they would accept that rats do need to stand (see Section 4.3 below). The fact that no respondents, even those in facilities with 20–23 cm caging, observed rats not being able to rear fully upright also demands further examination. This finding could be due to a number of factors, such as housing younger (therefore smaller) rats, the normalisation of postures that are not completely upright, and the fact that most workers did not observe rats at night, their most active period.

A fear of being seen as anthropomorphic, or being convinced by anthropomorphic arguments, frequently came to the fore. One researcher felt that animal technologists were anthropomorphic when they suggested refinements, stating that they were unable to provide data to substantiate these. However, there is a very well-established literature to justify providing the kinds of refinement listed by this respondent (facilitating hiding, chewing and foraging) [28,29,30,31,32,33,34]. Although some participants appeared to be getting ‘stuck’ on the concept of anthropomorphism, there is increasing recognition that *critical anthropomorphism* can be useful in identifying refinements to evaluate [35]. Thinking “if I were a rat I’d like that” can in fact be a useful starting point, provided that this assumption is then critically and empirically evaluated, using recognised animal welfare science techniques. However, leadership from motivated individuals or bodies is needed to move beyond the dismissal of suggestions as anthropomorphic to insist on an evaluation study, literature search or consultation with other users.

### 4.3. Effects on the Science

The researchers interviewed by HM were generally not concerned about potential impacts of larger, or higher, cages on scientific quality, with provisos relating to consistency amongst all the study animals and adequate comparisons with baseline data. Although there were some reservations about the need to validate and interpret data if these did prove to be altered, others saw this as an opportunity to improve the animal model, potentially leading to reduction as well as refinement. One researcher made the point that ‘things change’ in general, such as bedding, feed and animal suppliers, and these factors are taken into account.

As was the case for animal welfare concerns, some scientific concerns appeared to be based on assumptions that had not been validated. For example, researchers conducting behavioural studies of pain research and spatial processing respectively said they were ‘not sure about the impact’ and would ‘imagine’ there would be differences, such as neurobiological differences in the hippocampus that would affect data. However, it has been found that higher cages did not affect mechanical and thermal sensitivity tests used in pain research protocols [13]. Furthermore, a study on the effects of increased spatial complexity, including double height caging, reported no difference in hippocampus-dependent task performance (T-maze) and enhanced learning (for females only) in a non-spatial task (Novel Object Recognition) [36]. Although the latter demonstrates an effect of higher (and more complex) caging, some participants believed that a more stimulating environment would improve translatability, where rats are used as models for humans. The statement that ‘better welfare means better science’ was widely expressed by respondents.

### 4.4. Types of Evidence

We asked respondents specifically what kinds of evidence would convince them that rats need higher cages, with the aim of understanding why some establishments (and individuals) do accept the currently available evidence, while others do not. As mentioned above, ‘hard’ scientific evidence was required to change practice, notwithstanding the anecdotal evidence that was accepted to justify not making changes. In Table 2 we have listed the kinds of evidence cited by respondents, and identified publications that include these.

Evidence of the kind that people would find convincing is thus already available, so it should be a priority to raise awareness about existing studies and make their findings more accessible. For example, some participants suggested that Makowska and Weary’s (2016) article could be summarised into an infographic. It is interesting that participants did not mention preference, motivation or cognitive bias tests, suggesting a lack of knowledge regarding animal behaviour and welfare science techniques, including the kinds of parameters that are used to assess animals’ mental states and welfare needs. Responses like these suggest that the animal welfare science literature is not getting through to either animal technologists or scientists, which is a concern. In the UK, the Named Information Officer, other Named Persons and the AWERB are tasked with ensuring that staff have access to relevant, species-specific knowledge.

There also seemed to be a perception that evaluation studies would have to be done in-house, consuming resources, rather than implementing the results of research done by others. This was due to a number of factors, such as a desire to observe how the change functioned in the specific context and constraints of their particular facility, a desire to personally witness the evidence, and a scientific concern for the models used in external studies to be translatable to their own animal model. In the case of employees who had previously worked at a facility that had implemented the welfare change, however, they were able to bring translatable experience from their former place of employment.

An ambivalent attitude towards anecdotal evidence also came to light, in that several respondents (with different roles) had faith in the experience and empathy possessed by animal technologists and care staff. It is very positive to see the respect afforded to these staff, who as a body are a critically important resource in improving animal welfare and the quality of the science. However, it is essential that these staff are able to access good quality education, training and CPD with respect to animal behaviour and welfare science, in order to minimize uncritical assumptions about animal behaviour.

### 4.5. Behaviour Change

Although many respondents cited animal technologists as very progressive in pushing for change, others stated that they presented opposition to change. For example, Pharmaceutical A stated that animal technologists were very negative about higher cages until they had seen the welfare benefits for themselves. Parallels were drawn with the need to convince staff about the benefits of capturing mice in tunnels, or cupped hands, instead of by the tail. A “two-pronged” approach was suggested, involving a combination of providing evidence and simply instructing people as to what would be happening. As with intensive farming where stockpersons are central to the welfare of the animal [39], animal technologists can be key drivers of welfare changes in an animal research environment.

There are some insights on how to achieve behaviour change from other fields. Much has been written in the field of behavioural economics, at the intersection of economics and psychology, on the science of enacting behaviour change, from the ‘nudge’ economics of Thaler and Sunstein [40] to the Behavioural Insights Team department set up by the UK government [41]. Humans as decision-making agents are prone to certain cognitive biases that can help to explain some of the behavioural resistance encountered when enacting a welfare change, and therefore provide insights into ways of overcoming these biases. The resistance to change observed in the animal facilities visited could be a result of status quo bias, the human cognitive tendency to prefer already existing norms [42]. This can be overcome through the use of default options, the effectiveness of which have been demonstrated by several studies on dietary behaviour change, where the default menu option provided is healthy or meat-free, as well as studies on switching consumers to renewable energy tariffs [43]. Utilising default options in an animal research laboratory environment might involve providing researchers with already-enriched cages and fixed playpen exercise regimes. Much of the research conducted in the area of behaviour change and animal welfare concerns meat consumption, dietary change and farm animal welfare [44], however there is much potential for the use of behavioural ‘nudges’ in the field of laboratory animal welfare. Moreover, the Theory of Planned Behaviour Change emerging from psychology [45] states that an individual’s behaviour is shaped by their intention. Intention is shaped by attitudes, subjective norms (social pressures) and perceived behavioural control (belief about their ability to carry out the behaviour). In this instance, attitudes can be influenced by providing an appropriately weighted combination of scientific and anecdotal evidence, subjective norms can be affected by good practice of other facilities, and training to empower staff can shape perceived behavioural control.

### 4.6. Study Limitations

There are several limitations that affected the study. Firstly, the facilities who agreed to be involved in such a study, especially those who initiated contact following the call for participants, were predisposed to champion animal welfare changes and thus give favourable responses regarding increasing cage height. However, even in these facilities they were some more sceptical members of staff, enabling us to obtain a wide cross-section of opinions. Moreover, given the focus on rat cage height, we were unable to pay sufficient attention to other factors equally crucial for rat welfare beyond the project’s remit, such as cage complexity and enrichment. Another limitation to consider is the fact that the study did not include any animal suppliers, where most laboratory rats are bred and, as highlighted by one the participants, this is an area with great potential for large-scale change in laboratory rat welfare. Further studies could survey animal suppliers in order to understand the obstacles to increasing rat cage height in that highly space-constrained and intensive context. It is also important to note that this study relied on self-reported data from interviewees regarding which factors they would need to support higher cages, and therefore does not necessarily reflect the practical process of enacting change.

## 5. Conclusions

Approximately 1.6 million rats are used in the EU every year, including almost a quarter of a million used in the UK [46,47] and most of these are housed in cages that do not permit them to stand up. As described in the introduction, the current standard for rat cage height in the UK is based on four or five publications and ‘good current practice’ [4,12]. Looking again at two of the papers cited by the Council of Europe’s Group of Experts [8,9], taken with the four papers listed in Table 2 [13,14,15,16] and the good practice examples identified in the survey, we propose that there is sufficient evidence to justify increasing rat cage height to a minimum of 30 cm. This is already happening in some establishments, but not others, and it is important to understand the reasons for this.

There are clearly issues relating to scepticism about the need to rear up, lack of knowledge and erroneous beliefs about rat behaviour, low awareness of animal behaviour and welfare science, and concerns about appearing ‘unscientific’ and anthropomorphic. The cognitive dissonance of many respondents requiring hard science to back up the need for a refinement on the one hand yet accepting (and creating) anecdotes with respect to animal welfare needs on the other, was especially striking. It is therefore important to be cautious of inconsistencies in how evidence is valued and evaluated.

Some respondents expressed a wish for empirical evidence but were not always able to explain what this would comprise, which also reflects a lack of knowledge about rat behaviour and animal welfare science techniques. It is also still unclear whether establishments would be willing to accept evidence from the literature, or from evaluation studies in other facilities, or whether they would want to evaluate it themselves every time. Examples of higher cages successfully being used at other facilities were also mentioned by respondents within the pharmaceutical industry, and is recognised as permissible evidence by regulators [4].

The AWERB can play a pivotal role in helping to address knowledge gaps and issues of perception. Several of its tasks are relevant to this, including ‘supporting named persons and other staff dealing with animals on animal welfare, ethical issues and provision of appropriate training’, ‘promoting awareness of animal welfare and the 3Rs’ and ‘helping to promote a Culture of Care’ [48]. In particular, the AWERB could reflect upon whether there are effective channels for developments in animal behaviour and welfare science to come to establishments’ attention, via the committee itself and the Named Persons, especially the Named Information Officer (NIO). It is clearly essential that the NIO has access to the resources they need (including time) to research, retrieve and pass on information. The AWERB could also review how effectively the training for all staff involved in animal care and use covers animal biology, behaviour and welfare.

This is also an issue for training providers themselves to consider, including independent companies and doctoral schools. There are clear knowledge gaps with respect to animal behaviour and welfare needs, coupled with a lack of understanding about animal welfare science. Scientists are highly knowledgeable about specific fields, and there can be a danger of making assumptions about animals that are detrimental to both welfare and science. We are aware that training courses for doctoral students, animal technologists and care staff, and project and personal licensees always have many topics to cover in the allotted time. However, this issue is of such importance that it is worth considering ways of making training on behaviour and welfare more impactful and maintaining interest and awareness in other ways such as in-house newsletters and seminars.

Applied animal welfare scientists may also wish to consider the findings of this study when designing and reporting their work. Consulting stakeholders such as scientists, animal technologists, laboratory animal veterinarians and the regulator, with respect to the level and nature of evidence they would regard as convincing, may be helpful in producing publications that will be given due consideration when reviewing practice.

With respect to economic factors, there do appear to be health and safety issues if staff are expected to handle larger, heavier cages in the same way as smaller cages. If housing in cages that do not permit standing up causes distress to rats, then resources should be made available to rectify this. Economic factors are an inevitable practical consideration, and staff from Contract Research Organisations have explained that they are working in a highly competitive environment. However, in the UK and EU there is a legal requirement to minimize distress caused by animal accommodation, as well as in procedures. This would also accord with the many public-facing statements establishments made that ‘animal welfare is paramount’ and ‘we operate according to the highest welfare standards’.

It is of interest that some facilities are willing and able to house rats in higher cages, and others are not. Different approaches are currently being employed to refine rat housing, and even the commercially available double-height cages are still believed to be a compromise by some, as the floor area is reduced. On the basis of the literature and current good practice, is it time to have a complete rethink about the way rats are housed?

**Figure 2:**
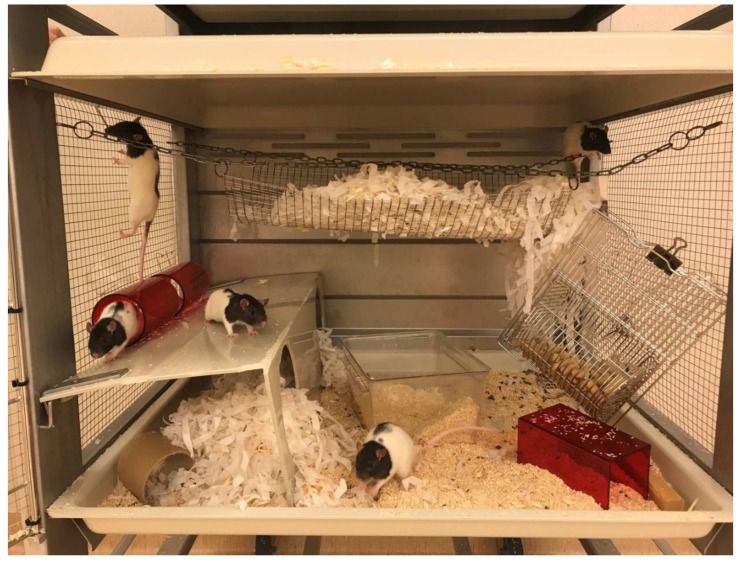
Refined rat housing which allows the animals to rear and climb. All the rats at RISE (Research Institutes of Sweden) are housed in modified rabbit cages. Photo: RISE.

As a thought experiment, we could consider how rat housing would look if legal minimum standards had to be designed from a blank slate, instead of the current situation in which a case had to be made to increase from a very low (literally!) baseline of a minimum cage height of 14 cm. As was the case when the last standards were set, there is an evidence base in the literature, with examples of good practice. If the standards were being defined today, would cages be significantly larger, with more than one level and high enough for all the rats to stand at once? This is already achievable, with appropriate infrastructure to deal with moving and cleaning the caging, as in the Research Institutes of Sweden shown in Figure 2. We can only speculate as to the counterinfluences that would be exerted by economic factors and perceptions regarding what would be a fair compromise between human and animal needs, and how all of these factors would be balanced. However, individual establishments can consider investing in better rat caging at any time, regardless of legal minimum standards, in accordance with their own Culture of Care.

We hope that this test case will lead to further discussions regarding the level and nature of evidence required to improve animal housing, husbandry and care, when it is appropriate to change engineering standards and mandatory Codes of Practice, and how to ensure that all staff receive adequate training in animal behaviour and welfare science.

## Figures and Tables

**Table 1:** Summary of recent research into the effects of upright standing on rat physiology and behaviour.

Publication	Summary of Study	Outcome and Authors’ Conclusions
Vachon (2014) [13]	Rats underwent either right sciatic nerve chronic constriction injury (CCI) or sham surgery; one month after surgery they were housed in cages 21 or 40.4 cm high.	Higher cages did not affect pain hypersensitivity (mechanical and thermal sensitivity tests), but anxiety was decreased and exploratory behaviours increased in higher cages. This strongly suggests that wellbeing increased.
Wheeler et al. (2015) [14]	Cognitive bias assay to evaluate the wellbeing of rats housed in a cage 40.4 cm high, with or without access to its full height.	Rats who were restricted to the bottom half of the cage, and then allowed to access the full height, demonstrated behaviour consistent with a positive affective state. Rats restricted to the lower half throughout the study had a tendency to increased neutrophil/lymphocyte ratios, an indicator of distress. Higher cages may improve rat well-being.
Dodelet-Devillers et al. (2016) [15]	Physiology and pharmacology of ketamine–xylazine anaesthesia on rats housed in cages 20.5 cm or 40.4 cm high.	Rats in higher cages recovered from anaesthesia faster and had lower heart rates. This was ascribed to increased activity benefiting the cardiovascular system. Relative adrenal gland: body weight ratios were greater for rats housed in the lower cages, suggesting a chronic increase in adrenocortical activity due to environmental-associated stress.
Makowska and Weary (2016) [16]	Study on the importance of burrowing, climbing and standing upright for laboratory rats. Recorded the propensity to burrow, climb and stand in 3, 8 and 13 month old rats, also compared frequency of compensatory lateral stretching between rats who could and could not stand upright. Rats were housed in standard cages (45 cm × 24 cm × 20 cm high) or semi-naturalistic cages with multiple levels (91 cm × 64 cm × 125 cm high).	Rats stood upright an average of 180 times a day at 3 months old (total standing time of 10 min), declining to 75 times a day at 13 months. Most upright standing is probably exploratory, so decrease with age may be due to reduced exploratory behaviour. All rats stretched laterally, but rats housed in standard height cages performed more lateral stretches to compensate both for the inability to stand and for the reduced mobility due to being housed in a smaller cage. Upright standing appears to be especially important to rats.

**Table 2:** Evidence that participants would find convincing with respect to increasing rat cage height.

Evidence	Studies That Have Included This
Biometric data, e.g., upright standing heights of different ages and strains of rat	An adult rat generally requires up to 30 cm to stand upright [37] and data from [8] indicate a maximally used height of 30 cm.
Observations over 24 h periods, recognising that rats are most active at night	Rats were filmed continuously for 24 h periods in [16]; between 16:00 and 18:00 (light period) and 22:00 and 0:00 (dark period) in [14]; and data were obtained during light and dark phases in [9].
Objective measures, e.g., of anxiety (presumably behavioural indicators) and levels of stress hormones, in standard versus higher caging	Decreased anxiety and increased exploratory behaviour demonstrated in higher cages [13] Behaviours consistent with positive affective state observed in higher cages [14]
Physiological data, e.g., on bone density and muscle growth	Making rats rise to an erect, bipedal stance for feeding, in cages 28–35.5 cm high, increased body and hind limb muscle mass, tibial bone mineral concentration and periosteal cortical bone formation in male SD rats [38] Increased activity benefiting cardiovascular system, evidenced by faster recovery from anaesthesia and lower heart rates [15]

## Appendix A

**List of Participants**

**Organisation**	**Cage Height**	**Interviewees**	**Notes**
CRO A 1	All 30 cm	1) Chairman/ELH 2) Regulatory Manager/NTCO/NIO 3) Facility Manager/NACWO 4) Animal Technologist	
CRO A 2	All 20–23 cm	1) Facility Manager 2) Animal Technologist	
University A	All 22 cm	1) Biomedical Research Facility Manager 2) Scientist/Researcher on neuropathic pain 3) NVS 4) Animal Technologist	
University B	Mixed: 20 and 34 cm	1) Interim Director of Biological Services 2) NACWO/Facility Manager/Department Safety Officer 3) AWERB Chair 4) NVS 5) Animal Technologist	
University C	Mixed: 21 and 26 cm	1) NACWO 2) Former Facility Manager/Former NACWO 3) Scientist 4) Health and Safety Advisor 5) AWERB Chair 6) Animal Technologist	
Pharmaceutical A	All 23 cm	1) Scientist/Researcher on 18 immune-inflammatory diseases 2) Health and Safety Officer 3) AWERB Chair 4) Former NACWO 5) Facility Manager 6) NIO	46 cm marmoset-style rat cages used at another of the organisation’s facilities.
Pharmaceutical B	Mixed: 20–23 cm and 30 cm	1) Director, In Vivo Sciences 2) Associate Director, In Vivo Sciences 3) Scientist/Researcher on in vivo pharmacology 4) Animal Technologist 5) Research and Development Manager, In Vivo Sciences	
Facility A	Mixed: 20–23 cm and 65 cm	1) Senior Animal Technologist 2) NVS 3) NACWO 4) AWERB Chair 5) Scientist	65 cm high marmoset-style bespoke cages.

## Appendix B

**Interview Guide**

*Facilities with 30 cm or higher cages:*

- What was the motivation for increasing cage height?
- Are you convinced, personally, that it was right to change practice and why?
- What was the most compelling evidence that convinced you?
- Were you aware of any human health and safety concerns associated with higher cages, before or after they were introduced?
- Were you aware of any financial/resource concerns with respect to changing to higher cages, and how were these overcome?
- Were you aware of any scientific concerns with respect to changing to higher cages, and how were these overcome?
- What in your experience are the main channels for information on developments in animal welfare science/good practice to come into the facility?
- Is the AWERB involved in sharing best practice or findings from research in animal welfare science?
- Are you aware of any negative outcomes associated with housing rats so they can rear upright, within this establishment? For example, on animal behaviour, scientific data, human health and safety, or resources?
- Have you noticed any changes in rat behaviour since the new cages were introduced?
- Have you been involved in studies to evaluate the welfare/scientific benefits of higher caging? If so, can you give us further details, including outcomes? Were these outcomes published?
- What do you personally think of the higher caging – does it meet the animals’ needs, or is further refinement required?

*Facilities with 20–23 cm cages:*

- Do you, personally, think the rat caging is high enough?
- Are you aware of the animal welfare concerns associated with 20 cm cages? Have you come across any scientific literature on this?
- Would you have any animal welfare concerns about introducing higher caging for rats?
- Are space and/or cost constraints a significant obstacle to introducing higher cages?
- Is there a process in place for people to request expenditure on the grounds that it would improve science and animal welfare? Has there been a precedent at this establishment, e.g., for environmental enrichment items?
- Do you have any human health and safety concerns about introducing higher cages?
- How would these health and safety issues be evaluated and evidenced? Has the issue of rat cage height arisen before?
- Do you have any scientific concerns about introducing higher cages?
- What are the main obstacles/issues to increasing cage height? Ideally, what would you do to overcome these (e.g., if resources/staff were unlimited)
- What kind of evidence, and how much evidence, would it take to convince you, personally, to support a change to higher cages and why?
- Have you observed any behaviours associated with impacts of cages that do not allow rats to rear upright (e.g., high levels of lateral stretching?)
- What in your experience is the main channel for information on developments in animal welfare science/good practice to come into the facility?
- Is the AWERB involved in sharing best practice or findings from research in animal welfare science?
- Do you feel that there is an effective mechanism for animal welfare science to be presented to the AWERB, and given due consideration? What is the channel for new information, and how is it discussed?
